# Similar recurrence rates among the 10 most used meshes for laparoscopic groin hernia repair: a nationwide register-based cohort study

**DOI:** 10.1007/s10029-025-03397-6

**Published:** 2025-06-27

**Authors:** Kristoffer Andresen, Mette W. Christoffersen, Jacob Rosenberg, Nadia Henriksen

**Affiliations:** 1 Department of Surgery, Copenhagen University Hospital - Herlev and Gentofte, Borgmester Ib Juuls Vej 1, Herlev, DK-2730 Denmark; 2 Center for Perioperative Optimization, Department of Surgery, Copenhagen University Hospital - Herlev and Gentofte, Borgmester Ib Juuls Vej 1, Herlev, DK-2730 Denmark; 3 Department of Surgery, Zealand University Hospital Køge, Lykkebaekvej 1, Køge, DK-4600 Denmark

**Keywords:** Groin hernia, Laparoscopic repair, TAPP, Mesh, Cohort study

## Abstract

**Purpose:**

Mesh-based repairs are the standard treatment for groin hernias. The effectiveness of mesh materials in laparoscopic groin hernia repair remains a concern and guidelines recommend a laparoscopic procedure with a heavy-weight mesh for adult patients. The objective was to assess the performance of the ten most used meshes for laparoscopic transabdominal preperitoneal (TAPP) groin hernia repairs in adults in regards to risk of recurrence.

**Methods:**

This nationwide register-based cohort study utilized data from the Danish Hernia Database, covering repairs from January 1, 2018, to June 30, 2023, from all surgical departments in Denmark. Patients operated with a TAPP repair with one of the ten most used meshes were included. The primary outcome was the cumulated risk of re-operation for recurrence, analyzed using Kaplan-Meier curves and Cox regression.

**Results:**

The study included 24,542 groin hernia repairs. All meshes showed a cumulated one-year re-operation rate below 1.5% and a five-year rate below 4%. Cox regression demonstrated lower Hazard Ratios for reoperation for some meshes, with several light-weight meshes having the lowest. For large inguinal hernias, no difference in cumulated re-operation rate was found.

**Conclusion:**

All ten of the most used meshes used for laparoscopic groin hernia repair in Denmark had less than 1.5% re-operation for recurrence within 12 months and less than 4% after 5 years. Lightweight meshes seemed to have good performance and it is not clear, even for large inguinal hernias, that a heavy mesh carries a benefit regarding the risk of recurrence, which contradicts current guidelines.

## Background

Groin hernia is a prevalent condition, with approximately 10,000 repairs performed annually in Denmark and an estimated 20 million globally [1]. Surgery is the treatment for groin hernias, and use of mesh is recommended in adults to reduce the risk of recurrence. The concept of using a prosthesis for hernia repair was first proposed in 1878 by Billroth [2], and since the 1960s polypropylene has been available for implantation in humans [2]. Mesh implants are important to ensure a long-term successful repair, characterized by a low recurrence and complication rate [3]. Given the high volume of procedures, the mesh market is substantial, valued at approximately US$ 2,133 million in 2023 [4]. This market is competitive, offering a variety of mesh products, differing in material (primarily polypropylene and polyester), weight (measured as g/m^2^), knitting style, and pore sizes [5].

The updated European Hernia Society guideline recommends that groin hernias are repaired laparoscopically with the use of a heavy-weight mesh (> 70 g/m^2^) [6]. Especially for large inguinal hernias, it is recommended to use a heavy-weight mesh to decrease the risk of recurrence. These recommendations were based on 12 randomized controlled trials with 2,909 patients. However, it remains controversial on which parameter the different mesh properties should be evaluated and the description of the mesh properties are heterogenous. Clinically, meshes are evaluated on outcomes such as recurrence of the hernia and postoperative complications such as infection, discomfort, and chronic pain.

One of the most important outcomes following groin hernia repair is recurrence rates as a recurrent hernia can result in the patient needing another surgery, that potentially is more complicated than the primary repair. The Danish Hernia database is a registry with nationwide coverage designed to monitor the quality of hernia repair by, among other outcomes, monitor re-operation rates for recurrences. The Danish Hernia database contains information on which meshes are in use and can therefore be used to monitor the performance of different meshes in regards to recurrence.

This study aimed to evaluate the performance of the ten most used meshes for laparoscopic inguinal and femoral hernia repair on a nationwide scale by specifically assessing the risk of re-operation due to recurrence.

## Methods

This was a nationwide register-based cohort study based on data from the Danish Hernia Database [7]. The study is reported according to the STROBE statement [8]. Since 1998, the Danish Hernia Database has recorded all inguinal and femoral hernia repairs, The purpose of the Danish hernia database is to monitor and improve hernia surgery in Denmark, and quality is monitored by several indicators [9]. Variables in the database include patient demographics such as age and sex, details regarding the hernia such as anatomy and size, and details regarding the operative approach such as laparoscopic or open surgery, including type of mesh [7]. Hernia size and location was classified according to the European Hernia Society classification for groin hernia, with L (lateral), M (medial), and F (femoral) depicts the anatomical location of the hernia, and where the number (1–3) relates to the size of the hernia orifice, i.e. M3 would be a medial or direct hernia, with an orifice of *≥* 3 fingers [10]. Information on mesh-weight was obtained from manufacturers’ websites and for those with a resorbable component we used the mesh weight after partial resorption of the mesh. According to a European Hernia Society Guideline, a mesh is considered heavy-weight when it is heavier than 70 g/m^2^ [6]. Reoperations for recurrence are captured by using the personal identification number given to all citizens of Denmark, allowing for cross-reference of registration in the database, should the patient have a subsequent repair irrespective of where in Denmark the reoperation is done.

This study included patients operated from January 1, 2018 to June 30, 2023. Inclusion criteria comprised elective procedures performed on adult patients, and it had to be an inguinal or femoral hernia repair using the transabdominal preperitoneal (TAPP) technique. TAPP is used in more than 98% of laparoscopic groin hernia repairs in Denmark [11]. Procedures, where the side of the hernia was missing was excluded, since it is paramount to know which groin was operated in order to investigate whether a subsequent operation was for a recurrent hernia in the same groin or if a subsequent hernia was located in the contralateral groin. Only patients undergoing TAPP repair with one of the ten most used meshes during the study-period were included. There was no access to procurement data for this study. The selection of type of mesh for groin hernia surgery in Denmark is up to the department that decides which meshes are available locally, and it is then up to the operating surgeon to decide which (of the locally available meshes) to use for their patients. Centers with a high re-operation rate was identified in the annual reports from the Danish Hernia Database [12]. Any center with an average re-operation rate *≥* 5% for the years 2018 to 2023 was excluded from analysis. This was done because centers tend to use one or few selected meshes, and thereby could skew the results when studying risk of re-operation following a specific mesh.

The primary outcome of this study was the cumulated risk of re-operation for recurrence. Re-operation for recurrence was used as a proxy for recurrence and defined as a subsequent hernia repair in the same groin, irrespective of the anatomical findings, meaning that a primary inguinal hernia repair that recurred as a femoral hernia was still counted as a recurrence since it resulted in a new operation for a hernia in the same groin. Curves of the Kaplan-Meier plots were compared using the log-rank test.

A multivariable Cox regression was conducted using the following covariates: Age (continuous variable), hernia anatomy (direct, indirect, etc.), side (left or right), type of mesh (commercial names), and hernia size (European Hernia Society classification) [13]. ProGrip™ mesh was used as reference because it was the largest group with a crude reoperation rate below 1%.

A subgroup analysis was done for large medial and lateral (M3/L3) hernias, as guidelines recommend using heavy-weight meshes especially for large hernias [6].

For statistical analysis and creation of Kaplan Meier plots, R version 4.2.2 (2022-10-31) within RStudio 2022.07.1, build 554, with the packages: dplyr, tidyverse, survival, ggplot2, and survminer, were used.

In Denmark, a formal approval from an institutional review board regarding ethics is not required nor possible for this type of study [14]. Permission was obtained from the Data Protection Agency (P-2023-14507), and the Regional Quality Assurance Program (DHDB-2023-07-08).

## Results

**Fig. 1:**
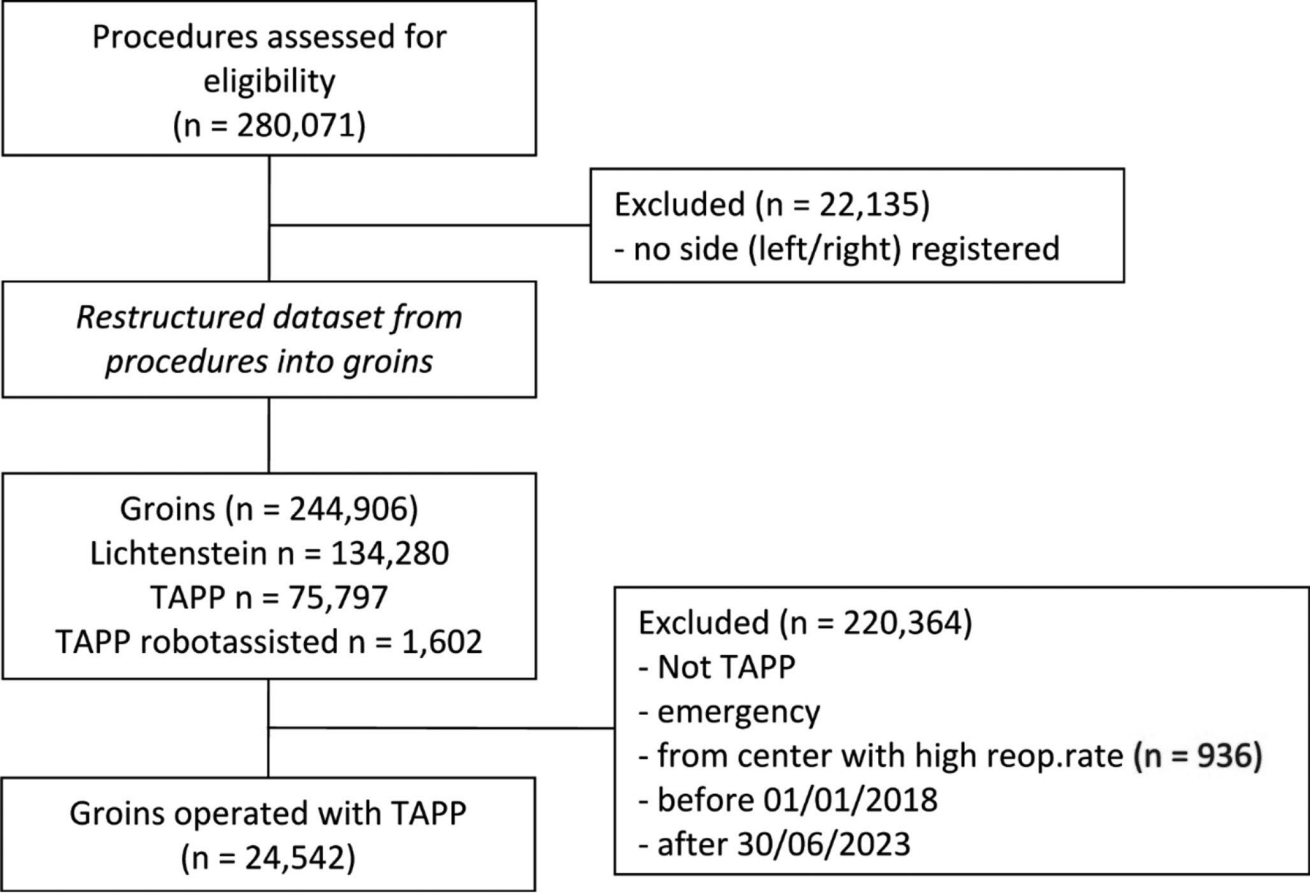
Flowchart

A total of 24,542 groins were included for analysis, see flowchart (Fig. 1). All patients from one private center were excluded due to a consistently high (*≥* 5%) 12-month re-operation rate for recurrence [9].

Included patients were around 60 years of age, with the majority being male, most were indirect inguinal hernias, and femoral hernias constituted less than 4% of the included repairs, for details see Table 1. The ten most commonly used meshes during the study period (January 1, 2018 to June 30, 2023) are listed in Table 2, being eight polypropylene meshes and two polyester meshes. Mesh weight varied between 30 g/m^2^ for the lightest and 80–100 g/m^2^ for the heaviest. Some meshes are regarded as self-fixating meshes, and some meshes are made from a mix of permanent and resorbable material. For all meshes, the crude, unadjusted re-operation rate for recurrence during the study period was less than 3%, Table 2.

**Table 1:** Demographics of included hernia repairs

TAPP	*N* = 24,542
Age, median (IQR) / mean (SD)	5 (48–70) / 58 (15)
Sex, Female/male, n (%)	3,477 (14) / 21,065 (86)
Side n (%)	
Left	10,898 (44)
Right	13,644 (56)
Anatomy n (%)	
Direct inguinal	7,969 (33)
Indirect inguinal	13,874 (57)
Pantaloon	1,126 (5)
Femoral	894 (4)
Other*	679 (3)
Size n (%)	
EHS1	6655 (27)
EHS2	14472 (59)
EHS3	3168 (13)
Unknown	247 (1)
Unilateral n (%)	18,225 (74)

*other includes femoral and inguinal combined and unspecified inguinal. EHS 1–3 refers to European hernia society classification for size of the hernia defect

**Table 2:** The 10 most commonly used meshes for TAPP from 2018 to 2023. Weight categorization based on manufacturers information

Name	*n* (%)	Reoperation rate *n* (%)	Material	Weight
3DMax™ light	2234 (9.1)	35 (1.6)	polypropylene	40 g/m^2^
Cousin	1808 (7.4)	18 (1.0)	polypropylene poly lactic acid*	30 ± 10 g/m² **
Dextile™	1124 (4.5)	3 (0.3)	polypropylene	> 90 g/m^2^
GalMesh	1072 (4.4)	16 (1.5)	polypropylene	80–100 g/m^2^
GalMesh light	1642 (6.7)	27 (1.6)	polypropylene	50 g/m^2^
Optilene^®^	3505 (14.2)	36 (1.0)	polypropylene	60 g/m^2^
Parietene™	5645 (23.0)	71 (1.3)	polypropylene	46 g/m^2^
ProGrip™	4877 (19.9)	35 (0.7)	polyester poly lactic acid*	38 g/m^2^ **
Ultrapro Advanced™	527 (2.2)	15 (2.8)	polypropylene poliglecaprone 25*	39 g/m^2^ **
Versatex™	2108 (8.6)	23 (1.1)	polyester	64 g/m^2^

*resorbable material, **weight after absorption

**Fig. 2:**
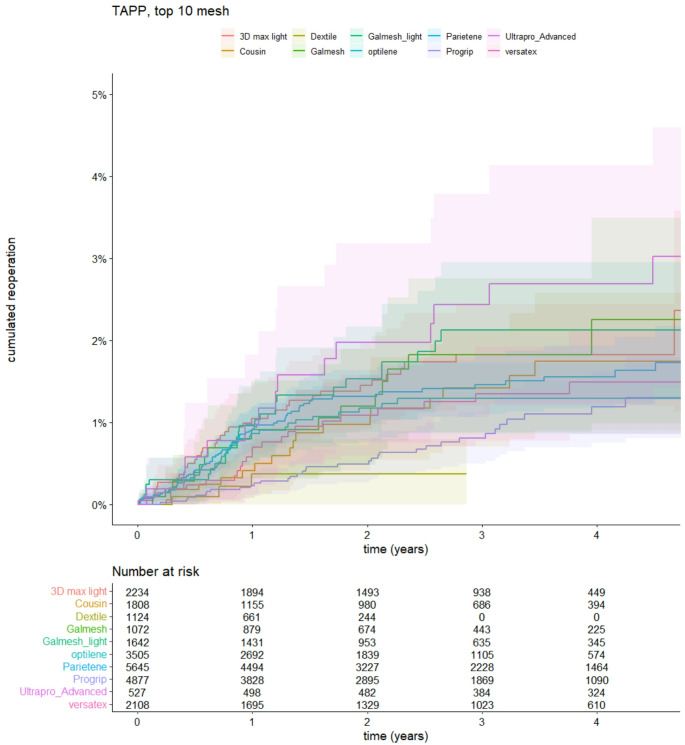
Kaplan-Meier curve illustrating the cumulated reoperation rates divided by the 10 most used meshes, including 95% confidence intervals. Log Rank, *p* = 0.046

The cumulated re-operation rate illustrated with the Kaplan-Meier curve is seen from Fig. 2. At one year, all meshes had a cumulated re-operation rate less than 1.5%, and after 5 years, all were still below 4%.

Significant differences between some meshes were found in the Cox regression, when adjusting for covariates such as age, side, hernia anatomy, and hernia defect size. The Parietene™ Mesh, 3DMax™ light, GalMesh Light, Optilene^®^, and Ultrapro Advanced™ had significantly higher hazard ratios for re-operation for recurrence compared with the ProGrip™ mesh, while there were no differences between ProGrip™ and Cousin, Dextile™, or Versatex™, Table 3.

**Table 3:** Cox regression, re-operation for recurrence as outcome

Covariates	Hazard ratio (95%CI)
Age	1.00 (0.99–1.01)
Hernia anatomy	
Inguinal - indirect	ref
Inguinal - direct	0.62 (0.47–0.82)*
Pantaloon	0.99 (0.59–1.66)
femoral	0.60 (0.28–1.29)
other	0.57 (0.23–1.38)
Side	
left	ref
right	0.92 (0.59–1.66)
Mesh	
ProGrip™	Ref
Parietene™	1.71 (1.14–2.56) *
3DMax™ light	1.96 (1.22–3.13) *
Cousin	1.57 (0.89–2.77)
Dextile™	0.54 (0.17–1.76)
GalMesh	1.76 (0.97–3.20)
GalMesh light	2.25 (1.36–3.74) *
Optilene^®^	1.50 (0.94–2.40)
Ultrapro Advanced™	2.75 (1.50–5.06) *
Versatex™	1.38 (0.81–2.34)
Hernia size	
EHS 1	ref
EHS 2	0.94 (0.69–1.28)
EHS 3	1.71 (1.16–2.51)
Unknown	0.97 (0.30–3.11)

CI: confidence interval. *p < 0.05. EHS 1–3 refers to European Hernia Ssociety classification for size of the hernia defect. All variables in the table were entered simultaneously into the model

Regarding the largest hernia defects (M3/L3), 2,761 groins were included in a subgroup analysis, including 1,084 direct and 1,677 indirect hernias, Table 4. There were no differences in the cumulated re-operation rate between the different meshes, *p* = 0.24, graph not shown.

**Table 4:** Subgroup for patients with a direct or indirect hernia, European Hernia Society (EHS) classification M3/L3

Mesh	*N*	Reop *n*(%)
ProGrip™	459	4 (0.9)
Parietene™	689	17 (2.5)
3DMax™ light	304	8 (2.6)
Cousin	152	5 (3.3)
Dextile™	81	0 (0)
GalMesh	284	5 (1.8)
GalMesh light	43	1 (2.3)
Optilene^®^	375	0 (0)
Ultrapro Advanced™	67	0 (0)
Versatex™	307	0 (0)
Total	2761	55 (2.0)

Reop = total unadjusted number of reoperations for recurrence

A sensitivity analysis including the center with high re-operation rates revealed that this center primarily used one type of mesh. When including the center, there were suddenly an unacceptable high recurrence rate related to one of the meshes, and it was clear that the vast majority of the recurrences following this mesh was from one center only.

## Discussion

The ten most commonly used meshes in Denmark for laparoscopic groin hernia repair had less than 1.5% reoperations for recurrence during the first 12 months following index surgery. All meshes had a cumulated 5-year re-operation rate of less than 4%. Regarding the largest defects, M3 and L3, there were no differences in the cumulated re-operation rate between the meshes. Light-weight meshes did not seem to result in a higher risk of reoperation for recurrence, even for the largest defects, contrary to the findings of the current guidelines.

The ProGrip™ mesh had a lower risk of reoperation for recurrence compared with Parietene™ Mesh, 3DMax™ light, GalMesh Light, and Ultrapro Advanced™. However, it was comparable to other meshes, such as Optilene^®^, Cousin, Dextile™, and the Versatex™ mesh. ProGrip™ has a self-fixating feature due to the small resorbable hooks built into the whole surface on one side of the mesh. The fixation is therefore atraumatic and resorbable. Other studies have also reported long-term low risk of recurrence using the ProGrip™ mesh [15]. Since the ProGrip™ mesh has small hooks on one side, a large dissection is required to place the mesh properly and good contact with the abdominal wall is needed for fixation. Furthermore, the fixation with small hooks means that the mesh is fixated over the triangle of doom and triangle of pain, which cannot be done when using traumatic fixation such as tacks. This means that a “standard” mesh with tack fixation has a risk of rolling up and away from the hernia defects when the peritoneum is closed and re-attached to the abdominal wall.

This study could not find a uniform association between the risk of reoperation and mesh weight, in line with a systematic review [16]. International guidelines recommend that a mesh with a weight of more than 70 g/m^2^ is used, especially for large defects such as the M3 and L3 hernias [6]. Only Dextile™ and GalMesh are considered to be heavy when using a cut-off of more than 70 g/m^2^. However, they did not perform better in this study, and there were no differences in the subgroup analysis of large M3/L3 hernias. The mesh categorization into light, medium and heavy weight covers a broad spectrum, and might be too simple [17]. Similarly, companies producing meshes use a large variation of classifications in their marketing material [5]. Other important factors that could influence the risk of recurrence include the mesh’s ability to adhere to the abdominal wall such as self-fixating meshes, the size of the mesh, ensuring that it is not too small, and the burst strength of the mesh.

Strengths of this study include that it is nationwide and with high external validity. All repairs, irrespective of being conducted by a supervised resident or an expert surgeon, were included. This study included large groups of patients for each mesh. Even the smallest group included more than 500 repairs. Registries are an optimal tool to evaluate mesh performance compared to smaller RCTs with selected patient groups and have previously shown useful [18, 19]. Therefore, it should be possible to detect clinically meaningful differences in risk of reoperation due to recurrence. Limitations of the study was that there was no patient reported outcome measures, and data regarding body mass index or smoking were not available. Surgeons do not register the ease of handling of meshes, and in this study, confounding factors such as the effect of supervision, surgeon experience, or mesh-fixation was not included. However, we believe that both known and unknown confounding factors are likely to be evenly distributed across the different types of meshes. Only patients with a recurrent hernia that received a re-operation for recurrence was registered as a recurrent hernia since there is no systematic clinical or radiological follow-up following groin hernia repair in Denmark. Therefore, the true recurrence rate is higher than the registered re-operation rate for recurrence, since not all recurrences will result in a re-operation for recurrence i.e. due to minimal symptoms or contraindications for re-operation. The re-operation rate therefore underestimates the true recurrence rate with up to 40%, accoding to a previous study [20]. However, we do not have reasons to believe that the type of mesh at index repair would affect the choice of performing a re-operation for recurrence and therefore, re-operation for recurrence is a valid proxy for recurrence, and a good outcome parameter when comparing meshes.

Mesh properties include size, weight, material, pore size, braiding technique, coating, self-fixating properties, shape and form, i.e. 3D preformed meshes and flat meshes. Regarding weight, the terms low- and heavy-weight are used, as presented in a recent systematic review including data from 48 randomized studies, meshes termed “low-weight” ranged from 35 to 60 g/m^2^, and meshes categorized as “heavy-weight” ranged from 72 to 116 g/m^2^. Interestingly, the pore size varied between 0.8 mm in diameter up to 4 mm in diameter, and overlapped between low- and heavy-weight meshes [17]. Another categorization of meshes is into flat-meshes and pre-shaped meshes, where the benefits of a pre-shaped mesh can be easier deployment and that the pre-shaped mesh does not need fixation [21]. These factors can also influence risk of recurrence; however, this was beyond the scope of the current study.

In perspective, these results demonstrate good long-term results of laparoscopic groin hernia treatment in Denmark. A nationwide database such as the Danish Hernia Database allows for continuous monitoring of quality and outcomes. Pitfalls when conducting register-based studies, including many centers, is that one center with one or few surgeons could be underperforming which may skew the results since centers tend to use one or few meshes. If a mesh is almost only used by an underperforming center, this can skew even nationwide data and wrong conclusions could be made. Therefore, it is important to have a good quality control mechanism like in the national clinical databases from Denmark. Sweden has similar demographics compared with Denmark and has a similar health care system. A cohort study of 25,190 laparoscopic groin hernia repairs found a total re-operation rate of 3.7%, which is similar to findings in this study. Furthermore, the study found that light-weight meshes did not increase the risk fo recurrence, when glue was used for fixation [22]. As the findings in this study is based on data from a clinical database with nationwide coverage, the external validity is high, as data reflects current practices.

In conclusion, all of the ten most commonly used meshes in Denmark had less than 1.5% reoperations within 12 months and also a 5-year cumulative reoperation rate of less than 4%. Lightweight meshes in this study seem to have a good performance and it is not clear, even for large inguinal hernias, that a heavy mesh carries a benefit regarding the risk of reoperation, which contradicts current guidelines.

## Declarations

### Data sharing

Raw data can not be made available.
